# Immunological signatures unveiled by integrative systems vaccinology characterization of dengue vaccination trials and natural infection

**DOI:** 10.3389/fimmu.2024.1282754

**Published:** 2024-02-20

**Authors:** Desirée Rodrigues Plaça, Dennyson Leandro M. Fonseca, Alexandre H. C. Marques, Shahab Zaki Pour, Júlia Nakanishi Usuda, Gabriela Crispim Baiocchi, Caroline Aliane de Souza Prado, Ranieri Coelho Salgado, Igor Salerno Filgueiras, Paula Paccielli Freire, Vanderson Rocha, Niels Olsen Saraiva Camara, Rusan Catar, Guido Moll, Igor Jurisica, Vera Lúcia Garcia Calich, Lasse M. Giil, Laura Rivino, Hans D. Ochs, Gustavo Cabral-Miranda, Lena F. Schimke, Otavio Cabral-Marques

**Affiliations:** ^1^ Department of Clinical and Toxicological Analyses, Faculty of Pharmaceutical Sciences, University of São Paulo, São Paulo, SP, Brazil; ^2^ Interunit Postgraduate Program on Bioinformatics, Institute of Mathematics and Statistics (IME), University of Sao Paulo (USP), Sao Paulo, SP, Brazil; ^3^ Departament of Immunology, Institute of Biomedical Sciences, University of São Paulo, São Paulo, SP, Brazil; ^4^ Department of Microbiology, Institute of Biomedical Sciences, University of São Paulo, São Paulo, Brazil; ^5^ Laboratory of Medical Investigation in Pathogenesis and Directed Therapy in Onco-Immuno-Hematology (LIM-31), Department of Hematology and Cell Therapy, Hospital das Clínicas, Faculdade de Medicina, University of São Paulo, São Paulo, Brazil; ^6^ Instituto D’Or de Ensino e Pesquisa, São Paulo, Brazil; ^7^ Fundação Pró-Sangue-Hemocentro de São Paulo, São Paulo, Brazil; ^8^ Department of Hematology, Churchill Hospital, University of Oxford, Oxford, United Kingdom; ^9^ Department of Nephrology and Internal Intensive Care Medicine, Charité University Hospital, Berlin, Germany; ^10^ Berlin Institute of Health (BIH) Center for Regenerative Therapies (BCRT) and Berlin-Brandenburg School for Regenerative Therapies (BSRT), Charité Universitätsmedizin Berlin, Berlin, Germany; ^11^ Osteoarthritis Research Program, Division of Orthopedic Surgery, Schroeder Arthritis Institute and Data Science Discovery Centre for Chronic Diseases, Krembil Research Institute, University Health Network, Toronto, ON, Canada; ^12^ Departments of Medical Biophysics and Computer Science, University of Toronto, Toronto, ON, Canada; ^13^ Institute of Neuroimmunology, Slovak Academy of Sciences, Bratislava, Slovakia; ^14^ Department of Internal Medicine, Haraldsplass Deaconess Hospital, Bergen, Norway; ^15^ School of Cellular and Molecular Medicine, University of Bristol, Bristol, United Kingdom; ^16^ Emerging Infectious Diseases, Duke-National University of Singapore (NUS) Medical School, Singapore, Singapore; ^17^ Department of Pediatrics, University of Washington School of Medicine, and Seattle Children’s Research Institute, Seattle, WA, United States; ^18^ Department of Medicine, Division of Molecular Medicine, Laboratory of Medical Investigation 29, University of São Paulo School of Medicine, Berlin, Germany; ^19^ Network of Immunity in Infection, Malignancy, Autoimmunity (NIIMA), Universal Scientific Education and Research Network (USERN), São Paulo, SP, Brazil

**Keywords:** dengue, vaccine, transcriptional signature, immune response, systems vaccinology

## Abstract

**Introduction:**

Dengue virus infection is a global health problem lacking specific therapy, requiring an improved understanding of DENV immunity and vaccine responses. Considering the recent emerging of new dengue vaccines, here we performed an integrative systems vaccinology characterization of molecular signatures triggered by the natural DENV infection (NDI) and attenuated dengue virus infection models (DVTs).

**Methods and results:**

We analyzed 955 samples of transcriptomic datasets of patients with NDI and attenuated dengue virus infection trials (DVT1, DVT2, and DVT3) using a systems vaccinology approach. Differential expression analysis identified 237 common differentially expressed genes (DEGs) between DVTs and NDI. Among them, 28 and 60 DEGs were up or downregulated by dengue vaccination during DVT2 and DVT3, respectively, with 20 DEGs intersecting across all three DVTs. Enriched biological processes of these genes included type I/II interferon signaling, cytokine regulation, apoptosis, and T-cell differentiation. Principal component analysis based on 20 common DEGs (overlapping between DVTs and our NDI validation dataset) distinguished dengue patients by disease severity, particularly in the late acute phase. Machine learning analysis ranked the ten most critical predictors of disease severity in NDI, crucial for the anti-viral immune response.

**Conclusion:**

This work provides insights into the NDI and vaccine-induced overlapping immune response and suggests molecular markers (e.g., *IFIT5, ISG15,* and *HERC5*) for anti-dengue-specific therapies and effective vaccination development.

## 1 Introduction

There is an urgent need for vaccines against neglected tropical diseases, such as helminth infections, Chagas disease, zika, and dengue fever (1–3). Among them, dengue poses a significant global health problem, spreading to new regions, including Europe (4). Several live-attenuated dengue vaccines are currently under research and development (3, 5–7), reflecting the ongoing efforts to combat this widespread infection. However, developing a dengue vaccine is still challenging due to the associated pathophysiology (5, 7, 8). Although some vaccines have been approved (9–11), the complex interaction between dengue serotypes and the human immune system represents a challenge for entirely safe and effective vaccines (12, 13). Hence, it is necessary to better understand the immunological mechanisms triggered by natural infection with DENV and their overlap with the immune response elicited by dengue vaccines, finding specific factors and signatures to monitor vaccine efficacy and guide the development of more potent vaccines (14, 15).

Dengue is among the most prevalent vector-borne diseases caused by the dengue virus (DENV), with an incidence of 100-400 million infections yearly (16, 17). Four dengue virus serotypes (DENV-1–4) have circulated throughout Asia, Africa, and the Americas. However, despite being a major global health problem, neither specific therapy nor a fully efficient vaccination protocol can ameliorate dengue’s destructive impact worldwide (3, 7, 10). While most infected individuals are asymptomatic (18), dengue can progress from a mild, self-limited disease called dengue fever (DF) to the more severe dengue hemorrhagic fever (DHF) during the defervescence phase (4, 19). In some individuals, the infection progresses to a life-threatening condition marked by acute vascular permeability, which is named dengue shock syndrome (DSS) (20). Therefore, a deeper understanding of the immunological mechanisms triggered by natural DENV infection is imperative. Moreover, elucidating how these mechanisms intersect with the immune response elicited by dengue vaccines could have far-reaching implications for vaccine design, effectiveness, and deployment strategies.

This study is part of the growing research area of systems vaccinology, which has successfully investigated the immune response to several viruses, for instance, influenza (21), yellow fever (22), and COVID-19 (23). So far, individual studies have characterized the immune response of dengue-infected patients or individuals enrolled in recent DENV vaccine trials (DVTs) (24–26). By employing an integrative systems vaccinology approach, our work aims to longitudinally characterize common immunological signatures between attenuated dengue virus infection models (herein called DVT) and their molecular overlap with natural dengue infection (NDI). Furthermore, we assessed this standard immunological signature in patients with different disease outcomes. Thus, through this comprehensive analysis, our work is part of a research field that has provided a global picture of the host response to vaccination, identifying potential immunologic signatures that can predict the immunogenicity of vaccines (27, 28). By understanding the interactions between natural infection and vaccine-induced immunity, we seek to provide new insights into dengue vaccine development.

## 2 Materials and methods

### 2.1 Curation of global gene expression data in dengue infection

We obtained publicly available transcriptomic data (RNAseq and microarray) from the Gene Expression Omnibus (GEO) (29) database. The studies included were selected using the following search terms: RNA sequencing, microArray, transcriptome, dengue, RNA sequencing, immune response, and “Homo sapiens”[porgn:_txid9606]. We included studies published between April 2011 and February 2022. Our inclusion criteria were: (1) studies with adult patients infected with DENV; (2) studies of whole blood (WB) or human peripheral blood mononuclear cells (PBMCs; (3) studies reporting disease phase and/or severity; (4) a minimum of 10 individuals per group. Our exclusion criteria were data sets that (1) included only children; (2) studies of *in vitro* infection; (3) studies that included additional flavivirus besides the dengue virus.

Additionally, we included one data set of a partially attenuated DENV-2 recombinant virus, which is part of a DENV vaccine challenge arm of a vaccine trial (DVT1, ClinicalTrials.gov NCT02021968) and three data sets for validation: 1 of natural dengue infection with different time points and severity groups (GSE43777) and two attenuated dengue vaccine trials: DENV3 trial (DVT2, ClinicalTrials.gov: NCT00831012; GSE98053) and tetravalent chimeric DENV2 (DVT3, ClinicalTrials.gov: NCT01224639; GSE146658). We obtained 955 transcriptome samples from ten data sets derived from either PBMCs or peripheral WB leukocytes described in detail in **Supplementary Table S1**, including exposure (primary or secondary infection, time-point, viral load, location, and serotype). After evaluating the study design, the number of samples, and other relevant information (disease phase and severity), we downloaded the transcriptome data sets and followed the analysis workflow of publicly available RNA-sequencing data sets as previously described (30). All R packages and bioinformatics web tools for this study are listed in the Key Resource Table (**Supplementary Table S23**).

### 2.2 Processing of RNA-seq data sets

Except for the data set GSE94892, which was available only as raw sequencing data and needed to be preprocessed as described below, all other transcriptomic data were available as non-normalized processed tables. The quality control of the raw and trimmed reads from the GSE94892 was performed using FastQC v.0.11.8 (31). Trimming of the adapter content and Quality trimming were performed using Trimmomatics v0.36 with the following settings: LEADING:20 TRAILING:20 SLIDINGWINDOW:4:25 MINLEN:31 (32). Kallisto v.46.0 (33) program was used to build the Kallisto index with reference transcriptome GRCh38 (Ensembl) with a k-mer length of 31 and to quantify abundances of the transcripts through the Kallisto pseudo-alignment, which provides estimates of transcript level counts. We used the tximport R package (34) to summarize count estimates at the gene expression level (**Supplementary Table S22**).

The raw cell counts for dataset GSE152255 were obtained in the original publication (26). Briefly, cell counts were obtained by merging BAM files per sample using SAMtools (35). Immune-cell proportions were analyzed with CIBERSORT (36) against LM22 gene signatures using the full expression matrix (Transcript Per Million, TPM >10). Tissue-specific signatures like mast cells and macrophages were excluded for whole blood application.

### 2.3 Differential expression analysis

Differential expression analysis (DEA) was applied to each dataset individually, followed by consensus analysis to identify common differentially expressed genes (DEGs) across the studies as previously described (37). We obtained DEGs for the microarray studies (GSE28405, GSE28988, GSE28991, GSE43777, GSE98053, and GSE94892) through GEO2R (38) and RNAseq data sets (GSE51808, GSE152255, and GSE146658) using Network Analyst (39), applying the limma-voom statistical method (40) as recently described (37, 41, 42). We applied the statistical cut-offs of adjusted *p*-value < 0.05, log2 fold-change (logFC) > 1 (up-regulated), or < -1 (down-regulated) to determine the DEGs. Comparisons established for each data set are available in the **Supplementary Table S2**. Considering substantial differences in the temporal collection points for each data set, we did not perform DEA by merging all datasets and correcting for batch effects. Instead, we performed a consensus analysis, treating individual studies as distinct and heterogeneous entities and searching for common gene signatures across the different studies.

### 2.4 Functional annotation and pathways enrichment analysis of overlapping genes

The intersection of (common) DEGs between each infection phase and disease severity was obtained using the web tool Intervene (https://asntech.shinyapps.io/intervene/) (43) and visualized by Upset graphics. We then performed functional enrichment of these overlapping genes across the data sets using Enrichr (https://maayanlab.cloud/Enrichr/) (44). In addition, functional enrichment of the intersection between each phase of natural infection was performed using the ClusterProfilerR package (45). The gene expression patterns of common DEGs were visualized through bubble heat maps with hierarchical clustering (applying the Euclidian distance metric) using the web tool Morpheus (https://software.broadinstitute.org/morpheus/) (46). Circos plots were obtained using the Circos web tool (http://circos.ca/) (47). An alluvial diagram was built to display the enrichment of the NDI overlap with DVTs of validation using the web tool SankeyMATIC (https://sankeymatic.com/) (48).

### 2.5 Random forest classification

We used random forest (RF) (49) to rank DEGs as predictors of dengue phases of the GSE43777 dataset using the R package random Forest (version 4.6.14) as previously described (42, 50). We applied the RF algorithm through five thousand trees, and the number of variables resampled was equal to three. Follow-up analysis used the Gini decrease, number of nodes, and mean minimum depth as criteria to determine variable importance. Interaction between pairs of variables was assessed using minimum depth as a criterion. The adequacy of the RF model as a classifier was evaluated by an out-of-bags error rate of less than 30% and receiver operating characteristic (ROC) curve analysis. For cross-validation with bootstrap aggregating, we split the data set into training and testing samples, using 75% of the observations for training and 25% for testing.

### 2.6 Principal Component Analysis and data visualization of stratifying genes

Principal Component Analysis (PCA) was performed with NDI-DVTs genes (log2 transformed values) that were DEGs in GSE43777, using the R functions prcomp and princomp, singular value decomposition (SVD) and eigenvalue decomposition statistics were applied. At the same time, biplots, clustering, and plotting were obtained through factoextra (51) and FactoMineR package (52). We also visualized DEGs belonging to NDI-DVTs gene sets in GSEs 43777, 51808, 94892, and 152255 through boxplots using ggplot2 package (53), applying the statistical Wilcoxon sum-rank test to compare the distributions of DF, DHF, and DSS pared independently. P values of less than 0.05 were considered significant as previously described (42).

### 2.7 Interactome analysis

For protein–protein interaction (PPI) analyses, we queried Integrated Interactions Database version 2021-05 (https://ophid.utoronto.ca/iid) (54) to obtain the network of interacting common DEGs between natural infection and vaccine challenge data sets, as well as the specific DEGs of each. We used all interaction sources. The network was annotated and visualized using NAViGaTOR 3.0.17 (55) and exported in an SVG file. The final figure was prepared with legends in Adobe Illustrator version 26.3.1.

### 2.8 Statistical analysis

For gene expression data, we applied a base 2 logarithmic function for each gene variable count data, herein called log2 gene expression or log2 intensity values, for bulk RNA and microarray sequencing, respectively. For the differential expression analysis, we applied the limma-voom pipeline, where empirical Bayes analyses are performed to identify differentially expressed genes. Adjusted P values of less than 0.05 were considered significant, and we applied the statistical cut-offs of log FC > 1 or < -1 to determine the upregulated and downregulated DEGs, respectively. We considered terms with adjusted *p*-values less than 0.05 significant for all enrichments.

## 3 Results

### 3.1 The longitudinal overlap between the immune responses observed during vaccination trials and natural dengue infections

**Figure 1:**
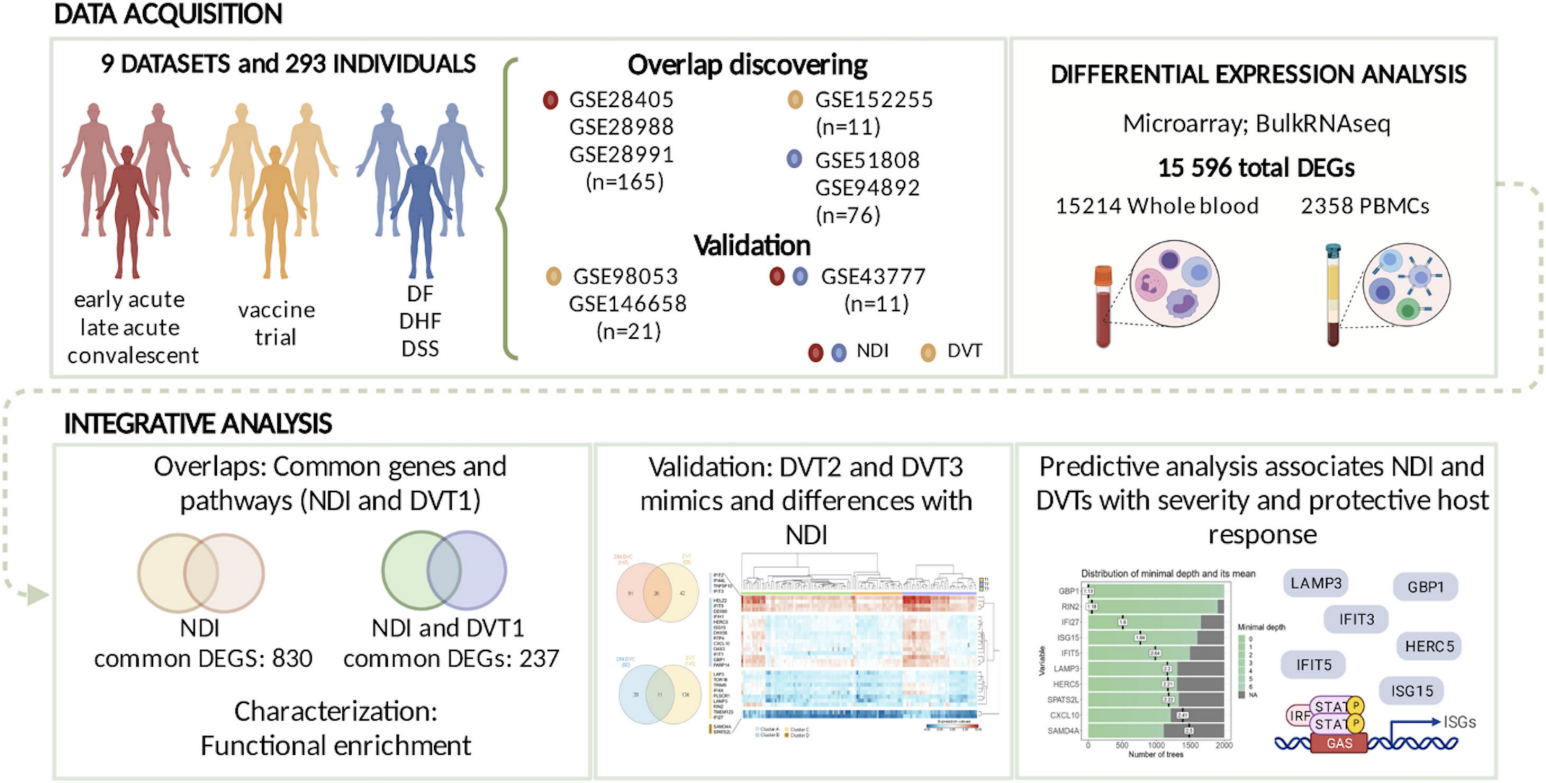
Study workflow. Overview of study workflow and results obtained. NDI, natural DENV infection; DVT, Dengue vaccine trial; DF, dengue fever; DHF, dengue hemorrhagic fever; DSS, Dengue Shock Syndrome.

We performed a comprehensive multi-study analysis of different dengue cohorts (**Figure 1**) to better understand immunopathogenesis and characterize immunological signatures that can establish the immunogenicity of vaccines. We obtained nine datasets of DENV infection transcriptomes according to our inclusion criteria described above. Concerning the serotypes investigated in the studies, our analyses encompassed all or nearly all serotypes within each dataset. This approach was taken because the NDI studies did not limit their analyses to only one serotype at a time (**Supplementary Table S1**).

**Figure 2:**
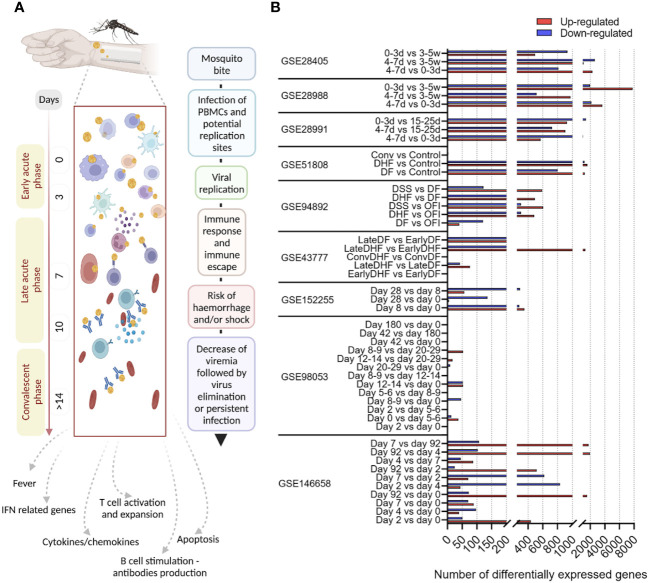
Dynamics of dengue infection and disease progression, and differentially expressed genes across the study cohorts. **(A)** Schematic overview of dengue phases and progression over time, showing the immunological processes and clinical manifestations^1–3^. **(B)** Graphic showing the number of differentially expressed genes (DEGs) by all data sets included in our study. The data sets are shown according to their Gene Expression Omnibus (GEO) IDs. Time points of sample collection and disease severity groups are shown for each data set according to the original studies.

By differential expression analysis, we identified DEGs across dengue infection cohorts of samples collected at different time points (**Figure 2A**; **Supplementary Tables S1–S3**). We found a consistently higher number of DEGs in the NDI data sets compared to the attenuated DENV infection studies (**Figure 2B**). From these DEGs, we performed further integrative systems biology analyses (**Figure 1**). It is worth noting the number of DEGs identified here differs from what was reported in the original publication of datasets (26, 56, 57) since we used a unique pipeline for our consensus DEG analysis applied to each dataset individually.

**Figure 3:**
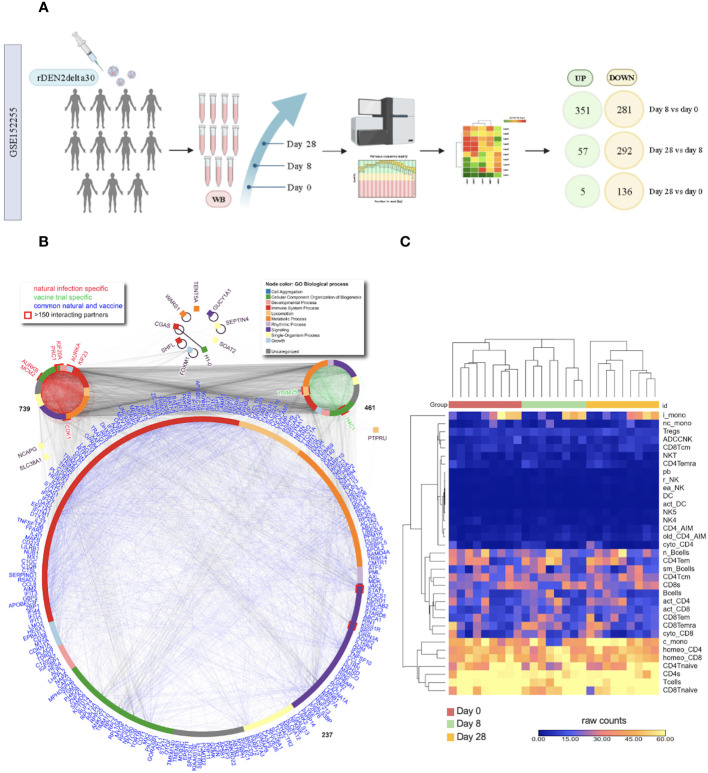
General transcriptional overlap between natural dengue infection and dengue vaccine trial. **(A)** The vaccine challenge design scheme shows the number of patients, days of sample collection, and the number of differentially expressed genes (DEGs) found in each comparison; **(B)** Protein-protein interaction network of common (overlap) DEGs between NDI and DVT1 (largest network) as well as specific DEGs of NDIs (in common across 3 NDI data sets) and the DVT1; **(C)** Heatmap of cell counts at different times of the DVT1 data set. Each column represents an individual, and rows indicate different cell populations. Bcells, B cells; pb, plasmablasts; sm_Bcells, switched memory B cells; n_Bcells, naïve B cells; c_mono, classical monocytes; i_mono, intermediate monocytes; nc_mono, non-classic monocytes; NKT, NK T cells; r_NK, resting NK; ea_NK, early activated NK; ADCCNK, ADCC NK; NK4, CD3–CD56intCD16– NK activated; NK5, CD3–CD56–CD16+ NK post-activation; DC, Dendritic Cells; act_DC, Activated DCs; Tcells, T cells; Tregs, regulatory T cells; CD4Tnaive, naïve CD4 T cells; CD4Tcm, central memory CD4 T cells; CD4Tem, effector memory CD4 T cells; CD4Temra, effector memory re-expression CD45RA CD4 T cells; homeo_CD4, homeostatic CD4 T cells; cyto_CD4, cytotoxic CD4 T cells; act_CD4, activated CD4 T cells; CD8Tnaive, naïve CD48 T cells; CD8Tcm, central memory CD8 T cells; CD8Tem, effector memory CD8 T cells; CD8Temra, effector memory re-expression CD45RA CD8 T cells; homeo_CD8, homeostatic CD8 T cells; cyto_CD8, cytotoxic CD8 T cells; act_CD8, activated CD8 T cells; CD4_AIM, AIM+ CD4 T cells; old_CD4_AIM, old AIM+ CD4 T cells.

The DVT1 study (GSE152255; DENV2; ClinicalTrials.gov: NCT02021968), a human dengue vaccine challenge trial using the partially attenuated dengue serotype 2 (rDEN2Δ30) virus, included 11 individuals and provided transcriptome samples from day 0 (before infection), days 8, and 28 after attenuated infection. When comparing the different time points, DVT1 had 413 upregulated (351 on day 8 vs. day 0; 57 on day 28 vs. day 8; 5 on day 28 vs. day 0) and 709 downregulated DEGs (281 on day 8 vs. day 0; 292 on day 28 vs. day 8; 139 on day 28 vs. day 0) (**Figure 3A**; **Supplementary Figure S1**; **Supplementary Table S14**). After excluding overlapping genes across different timepoints, we identified 411 upregulated and 623 downregulated DEGS. We found 237 common DEGs during the immune response against the attenuated dengue virus (DVT1 data set, GEO152255) compared to the NDI data sets that lacked severity information (GSE28991, GSE28988, and GSE28405). These overlapping DEGs are highly associated with and enrich mainly in biological processes (BPs) related to the immune system response, metabolic processes, and cellular component organization of biogenesis. We found 739 and 461 DEGs in the NDI and DVT1 data sets, respectively, which enriched mainly similar BPs (**Figure 3B**). Overall, there were no striking differences in the distribution of cell numbers along with the DVT1 time points, i.e., there are similar cell frequencies, displayed as raw counts, across time (**Figure 3C**), underscoring the transcriptional changes during the immune response against the attenuated dengue virus.

**Figure 4:**
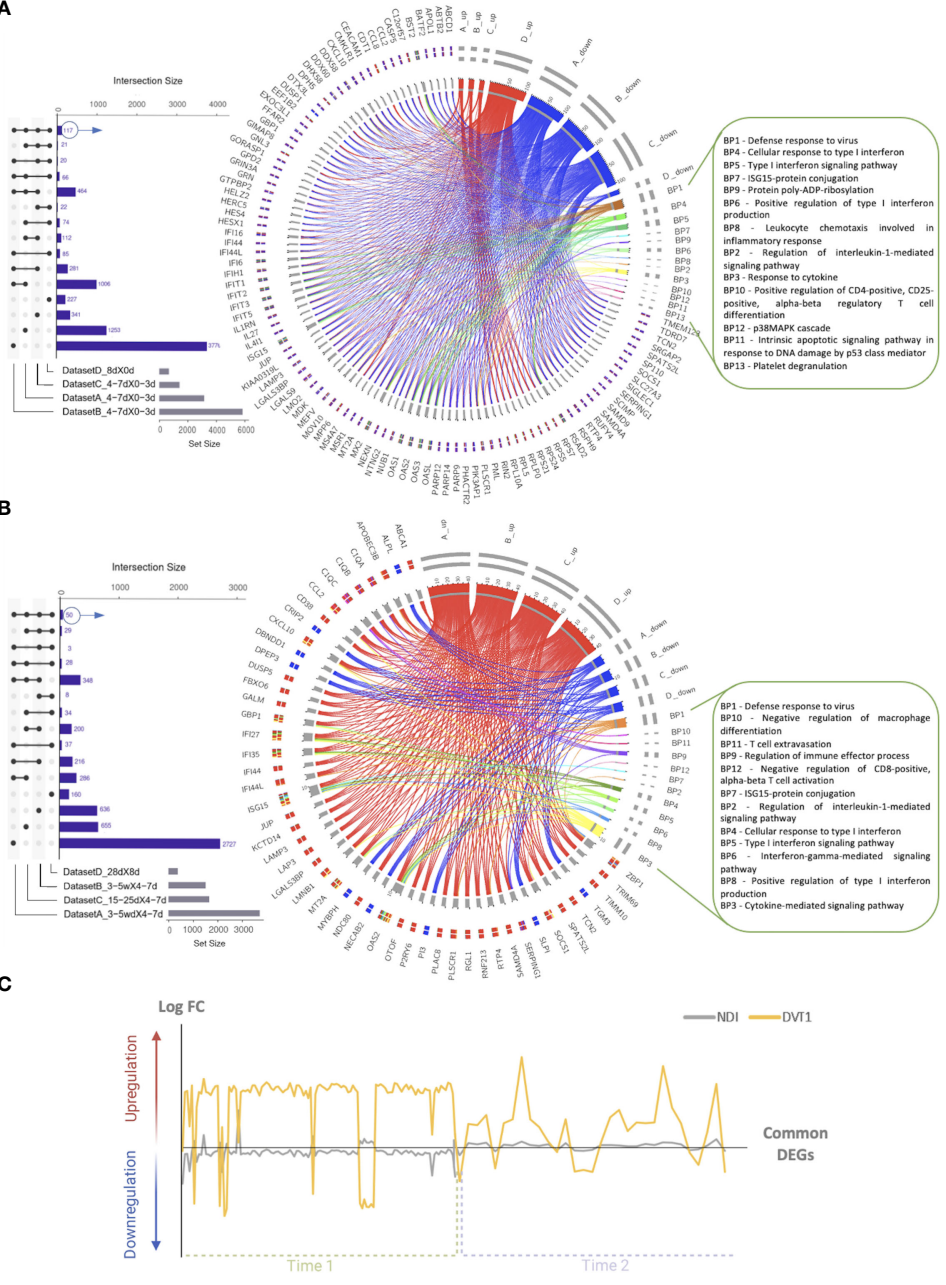
Longitudinal transcriptional overlap between the natural dengue infection and the dengue vaccine trial. Upset plots displaying overlapping differentially expressed genes (DEGs) from the comparison of the **(A)** initial (total of 117 DEGs; only 100 are exhibited in the circos plot) or **(B)** late (total of 50 DEGs) days of sample collection in NDI data sets [**(A)** GSE28405, **(B)** GSE28988, **(C)** GSE28991] and the DVT1 data set [**(D)** GSE152255]. Circos plots illustrate the functional relationships (shown by edges) between the DEGs and biological processes (BPs), denoted by letters. Colors denote up- (red) and downregulation (blue) of DEGs. The complete list of enriched BPs is provided in **Supplementary Tables S4A**, **S5**. **(C)** The graphic provides an overview of whether DEGs are up or downregulated (by relative log FC on the y-axis) during the initial (Time 1) or late (Time 2) days of sample collection. Variables = genes.

Of note, during the initial phase (time 1) of sample collection, we found 117 overlapping DEGs (**Figure 4A**) between the DVT1 (GEO152255) and NDI (GSE28991, GSE28988, and GSE28405) data sets. However, those DEGs were often regulated in the opposite direction (DEGs upregulated in DVT1 are downregulated in NDI and the other way around) (**Figure 4C**). While this regulation variance was prominent in time point 1, we found at time point 2 a more constant common upregulation of DEGs between NDI and DVT1. A possible explanation for this observation consists in the fact that while the DVT1 study compared samples collected during the acute (day 8) infection with those obtained before infection (day 0) and day 8 versus convalescence (day 28) after infection, all NDI data sets have comparisons between late (days 4-7) versus early (days 0-3) as well as acute and late acute versus convalescent (2-5 weeks) phases. Hence, since these studies have different start points of sample collection, future investigations require longitudinal comparisons synchronizing study time points. Nevertheless, these 117 overlapping DEGs enrich 13 most relevant BPs, based on *adjusted P-value*, excepted redundant BPs, including defense responses to the virus, platelet degranulation, and several interferon (IFN)-related BPs (**Figure 4A**; **Supplementary Table S4**).

At the late time point of sample collection (DVT1: day 28 *vs.* day 8; NDI: 2-5 weeks *vs*. days 4-7 [time 2] of NDI), we found a transcriptional overlap of 50 DEGs (**Figure 4B**) between DVT1 (GEO152255) and NDI (GSE28991, GSE28988, and GSE28405). Most common DEGs were longitudinally upregulated in DVT1 and NDI (**Figure 4C**). Functional enrichment analysis of these 50 DEGs resulted in 12 statistically significant gene ontology BPs (**Figure 4B**; **Supplementary Table S5**), ranging from defense response to the virus (the most significantly enriched BP) to negative regulation of CD8-positive/alpha-beta T cell activation. Among these BPs, there are also several interferon-related signaling pathways.

### 3.2 Interferon-associated signature marks the overlap of the acute phase in severe natural dengue infection and the vaccination trial

**Figure 5:**
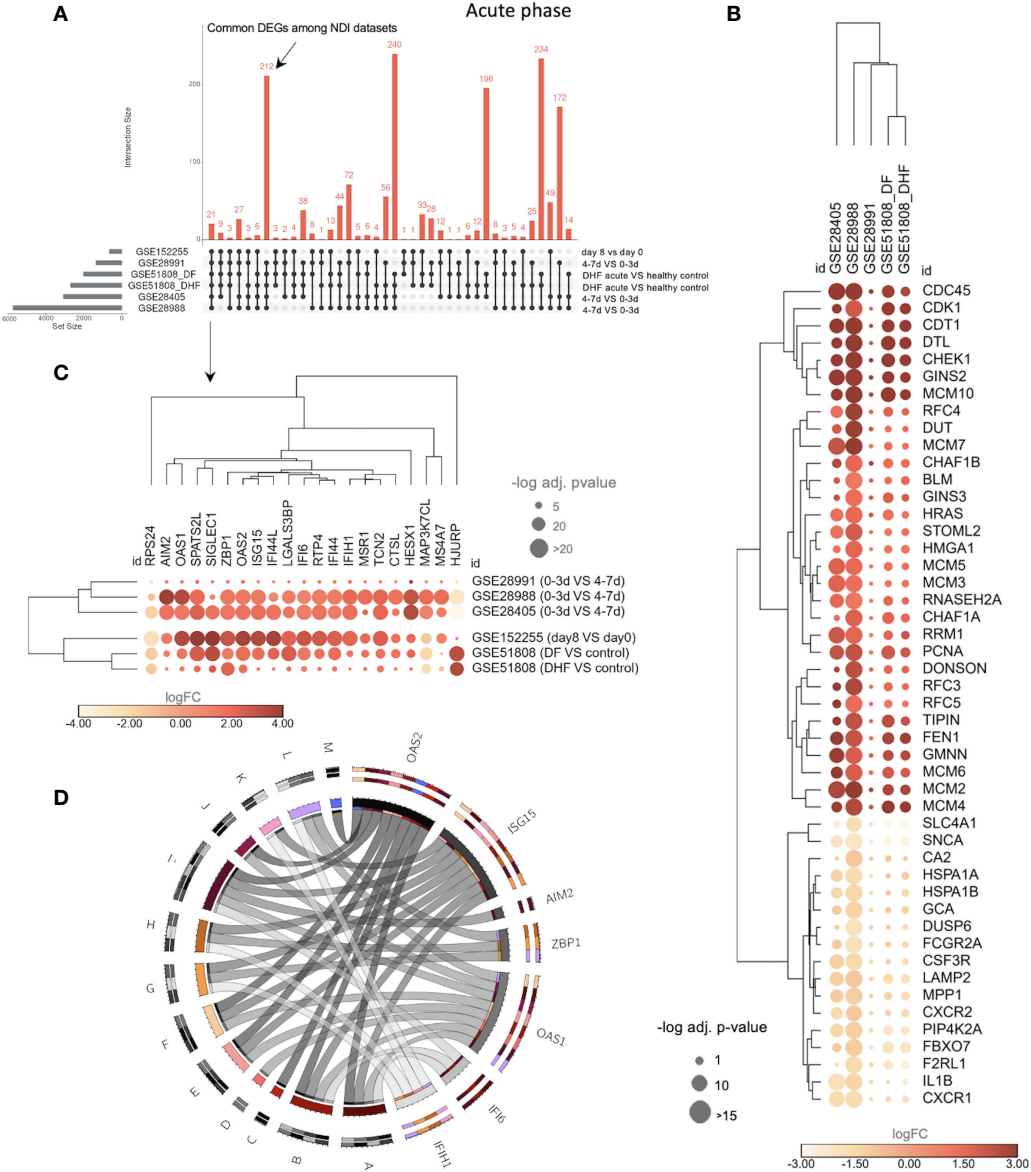
The transcriptional overlap between NDI at acute phase with disease severity and DVT1 information. **(A)** The upset plot showing the transcriptional intersection among all datasets of the NDI acute phase is as follows. GSE28988 and GSE28405: late acute (4-7 days or d) vs. early acute (0-3d); GSE51808: DF or DHF acute (2-9d) vs. healthy controls; GSE28991: late acute (4-7d) vs early acute (0-3d); GSE152255 (DVT1): first days of infection (8d) vs (day 0). DF, dengue fever; DHF, dengue hemorrhagic fever. The dataset GSE94892 was not included in this comparison because there is no information on time points for the different disease severity states (DF, DHF, and DSS) (**Supplementary Table S6**). **(B)** Bubble heatmap showing 48 common genes enriching the top 11 (1 term, 31 genes, from upregulated common DEGs enrichment and 10 terms, 17 genes, from downregulated common DEGs enrichment, based on adjusted p*-*value and number of genes, **Supplementary Tables S7, S8**) gene ontology (GO) biological processes (BP) among the NDI datasets. **(C)** Bubble heatmap of 21 common genes across all datasets. The color of the circles corresponds to log2 fold change (log2FC), and their size is proportional to the -log10 of the adjusted p-value. **(D)** Circos plot indicating the relationships between 7 of the 21 genes [shown in **(C)**] and statistically significant BPs (denoted by letters) resulting from enrichment analysis of the 21 genes using Enrich R^4^. The size of the rectangles in the outer circles is proportional to the involvement of each gene in multiple pathways. The size of rectangles forming the inner circle represents genes and pathways with more connections to each other. Colors (chosen randomly to discriminate each variable) on the outer circles denote pleiotropy and gene-pathway associations. A, cellular response to type I interferon; B, type I interferon signaling pathway; C, regulation of nuclease activity; D, negative regulation of viral genome replication; E, negative regulation of viral life cycle; F, regulation of viral genome replication; G, regulation of type I interferon production; H, regulation of cytokine production; I, cytokine-mediated signaling pathway; J, regulation of RNA metabolic process; K, negative regulation of type I interferon production; L, positive regulation of type I interferon production; M, interferon-gamma-mediated signaling pathway. The complete list of enriched BPs is provided in **Supplementary Table S9**.

To further understand the DVT1 and NDI intersection, we characterized the common DEGs, including data sets with acute dengue phases (late x early acute, GSE28991, GSE28988, and GSE28405) and disease severity (GSE51808: DF and DHF) information (**Figure 5A**). We found 212 common DEGs between the NDI data sets (**Figure 5**; **Supplementary Table S6**). Among them are 164 upregulated DEGs, enriching cell cycle-associated BPs such as DNA replication, DNA conformation change, chromosome segregation, and cell cycle checkpoints (**Supplementary Figure S2A**; **Supplementary Table S7**). Furthermore, there were 48 common downregulated DEGs, enriching BPs such as myeloid cell differentiation, negative regulation of phosphorylation, neutrophil degranulation leukocyte chemotaxis, and neutrophil migration (**Supplementary Figure S2B**; **Supplementary Table S8**). Consequently, these BPs are upregulated at the beginning of the infection. **Figure 5B** displays the gene expression profile of the 48 DEGs associated with the most significantly enriched BPs, either up (the first most enriched) or downregulated (the ten most enriched) (**Supplementary Tables S7, S8**). These DEGs are expressed in the same direction, i.e., consistently upregulated or downregulated across the NDI data sets.

Using the same data sets across NDI and DVT1 sets (GSES 28405, 28988, 28991, 51808, and 152255), we found a total of 21common DEGs (**Figure 5C**; **Supplementary Table S6**). These 21 DEGs enrich BPs such as type I IFN-related BPs (e.g., cellular response to type I IFN, type I IFN signaling pathway, regulation of type I interferon production) as well as regulation of cytokine production, cytokine-mediated signaling pathway, negative regulation of the viral life cycle, and regulation of RNA metabolic processes (**Figure 5D**; **Supplementary Table S9**), indicating a consistent interferon-associated signature that marks the overlap of the acute infection of NDI and DVT1.

### 3.3 The transcriptional response during severe natural infection differs from that of the attenuated phase when the host returns to immune homeostasis

**Figure 6:**
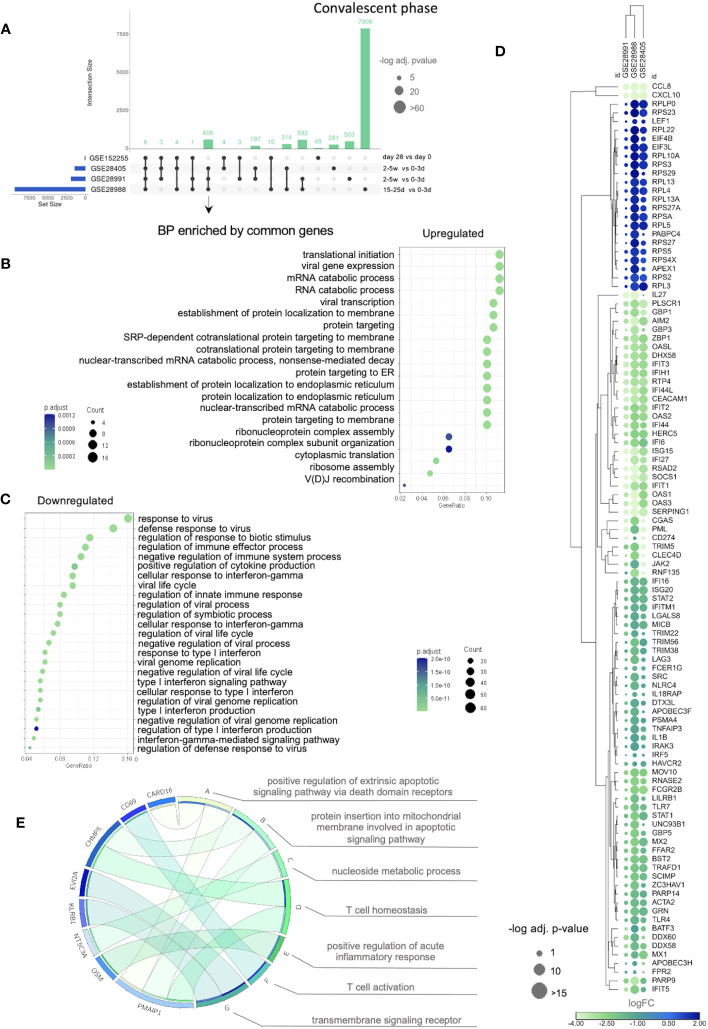
The transcriptional divergence between NDI and DVT1 during the convalescent phase. Data sets (GSE28405, GSE28988, and GSE28991). Since the GSE51808, which contains information regarding disease severity, did show only four DEGs during the convalescent phase, it was not included here. **(A)** Upset plot shows the intersections between datasets of the convalescent phase from the NDI data sets (DEGs obtained from the comparison between 3-5 weeks *vs*. 0-3 days) and the vaccine trial (DVT1) (sample collected 28 vs 0 day) (**Supplementary Table S10**). **(B)** Dotplots of BPs enriched by upregulated (**Supplementary Table S11**) and **(C)** downregulated common genes (**Supplementary Table S12**) across NDI datasets. **(D)** Bubble heatmap showing clusters of up- and downregulated genes enriching the most significant BPs shown in **(B, C)** (based on adjusted p*-*value and number of genes). The circle sizes and color are proportional to the log fold change (log FC) and -log10 adjusted p-value (-log10 adj. p-value), respectively. **(E)** Circos plot indicating the relationship between the 8 common DEGs across all NDI and DVT1 data sets [shown in **(A)**] and statistically significant BPs enriched by these genes. The complete list of enriched BPs is provided in **Supplementary Table S13**.

We next evaluated the transcriptional intersection between the DVT1 and NDI at the convalescent phase compared to day 0 in DVT1 and the early acute phase in the NDI data sets. We found 606 common DEGs between the NDI data sets (GSE28405, GSE28988, and GSE28991) (**Figure 6A**; **Supplementary Table S10**). During the convalescent phase, upregulated DEGs across all NDI datasets enriched several regulatory mechanisms involved in protein production and RNA expression (**Figure 6B**; **Supplementary Table S11**). Meanwhile, functional enrichment analysis of downregulated DEGs suggested the return of the immune system to its homeostatic phase after more than two weeks (**Figure 6C**; **Supplementary Table S12**). **Figure 6D** displays the expression pattern and clusters of common upregulated and downregulated DEGs, enriching the most significantly affected BPs (**Supplementary Tables S11, S12**).

Of note, only eight DEGs from the DVT1 data set overlapped with all NDI data sets. This small number of overlapped genes is expected once DVT1 compares day 0 as pre-infection, where the immune response had not been initiated yet, while NDI compares early and late acute phases (**Figure 6E**; **Supplementary Table S13**).

Despite having a small number of common DEGs, we found several common BPs between the DVT1 and all NDI data sets, enriched by the total number of DEGs from acute phase comparisons of these studies (**Supplementary Figure S3A**; **Supplementary Table S15**). For instance, these BPs include cytokine-mediated signaling pathways, type I interferon signaling pathways, interferon-gamma-mediated signaling pathways, and inflammatory responses. These findings suggest that the immune responses to attenuated dengue virus and natural dengue infection elicit similar functional processes during the acute phase. During the convalescent stage, there were less common BPs, e.g., cytokine-mediated signaling pathways, cytoplasmic translation, and RNA processing), suggesting different returns to immune homeostasis of DVT1 and NDI. (**Supplementary Figure S3B**; **Supplementary Table S16**).

### 3.4 Different dengue vaccination trials share common DEGs with the natural dengue immune response

**Figure 7:**
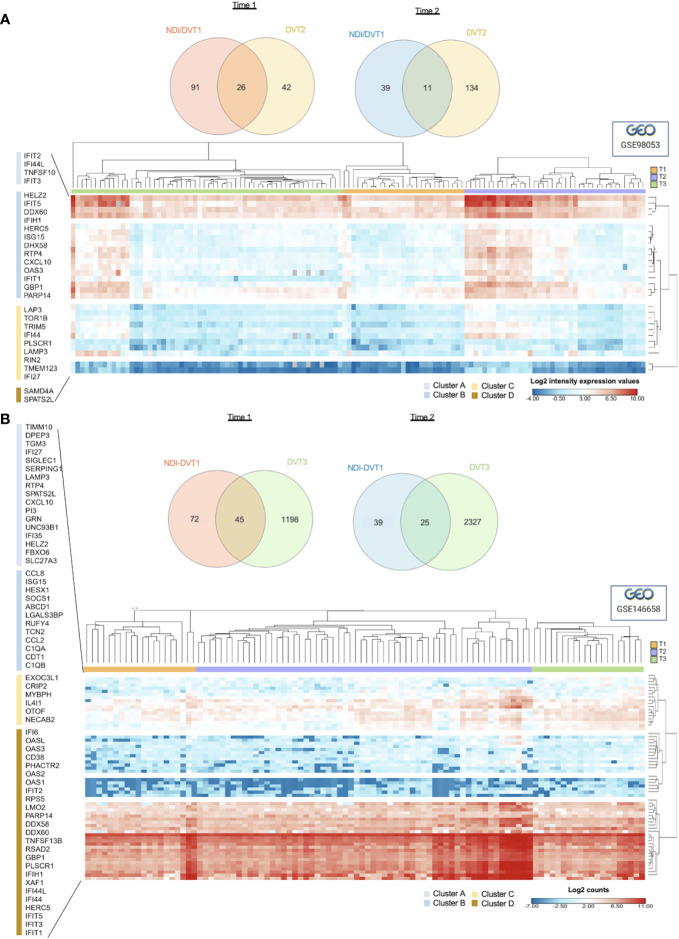
Validation of transcriptional overlap and immune response dynamics between NDI and DVT1 using two additional vaccine trials (GSE98053 and GSE146658). **(A)** Venn diagrams (upper graphics) showing the intersections of common differentially expressed genes (DEGs) from NDI/DVT1 and DVT2 datasets at acute (Time 1) and convalescent phases (Time 2). Complex heatmap (lower graphics) with hierarchical clustering (using Euclidian distance metric) of expression intensity values (GSE98053) of 28 genes that overlap between NDI/DVT1 and DVT2 data sets through time 1 (T1, from 0 to 5 days), time 2 (T2, from 6 to 9 days), and time 3 (T3, 12 or more days), respectively, resulting in 4 clusters (Cluster A to D). **(B)** Venn diagrams (upper graphics) showing the intersection of common DEGs from NDI/DVT1 and DVT3 data sets at acute (Time 1) and convalescent phases (Time 2). Complex heatmap (lower graphic) with hierarchical clustering (using Euclidian distance metric) of log2 counts (GSE146658) of 60 genes that overlap between NDI/DVT1 and DVT3 data sets at different time points as described in **(A)**.

To further explore the overlap between the DVT1 and NDI data sets, we assessed the intersection of these DEGs in the other 2 DVT trials, indicated here as DVT2 (GSE98053; DENV3; attenuated dengue vaccine rDEN3Δ30/31–7164, TV003; ClinicalTrials.gov: NCT00831012) and DVT3 (GSE146658; DENV-2-1, DENV2, DENV2-3, DENV2-4; attenuated dengue virus serotype-2 strain, DENVax-2; ClinicalTrials.gov: NCT01224639). **Supplementary Figure S4** illustrates the design of DVT2 and DVT3 studies. The DVT2 data set presented 26 and 11 common DEGs at acute (Time 1) and convalescent phases (Time 2), respectively, with the NDI/DVT1 data sets, resulting in 28 unique common DEGs (**Figure 7A**; **Supplementary Table S17**). These genes form four expression clusters, which enrich several BPs related to IFN, anti-viral, and cytokine-mediated immune responses (**Supplementary Figure S5A**; complete enrichment in **Supplementary Table S18**). The DVT3 dataset shared 45 and 25 common DEGs with NDI/DVT1 at times 1 and 2 (**Figure 7B**; **Supplementary Table S19**), respectively. These genes enrich related biological processes compared to the NDI/DVT1-DVT2 overlap (**Supplementary Figure S5B**; complete enrichment in **Supplementary Table S20**).

### 3.5 DEGs distinguishing dengue severity are consistently regulated across dengue vaccination trials

**Figure 8:**
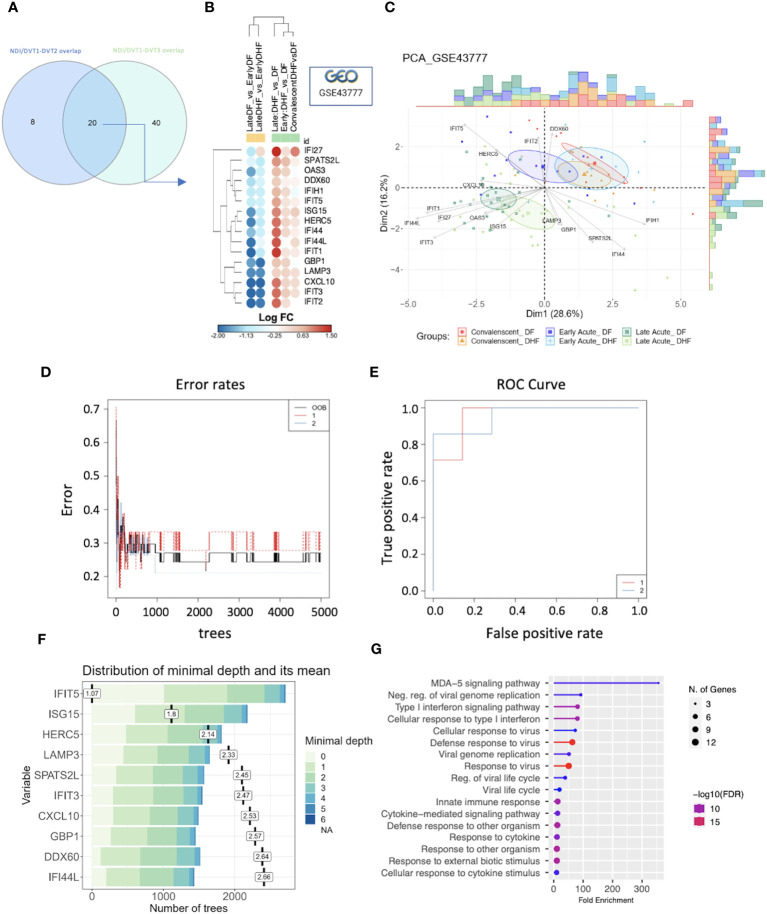
GSE43777 validates NDI-DVTs overlap and predicts important genes for distinguishing disease severity and establishing host protection through a machine learning model. **(A)** Venn diagram showing the intersection (20 genes) between common genes from NDI/DVT1-DVT2 and NDI/DVT1-DVT3 overlaps. **(B)** Bubble heatmap with hierarchical clustering using Euclidian metric of 16 of these 20 genes, which were differentially expressed genes (DEGs) when comparing groups of disease phases (yellow cluster) and severities (green cluster) of the data set GSE43777. The colors of the circles are proportional to log FC, indicating downregulation(blue) or upregulation(red) of each gene. **(C)** Principal component analysis (PCA) from the 16 DEGs stratifying late acute phases from the other disease phases (n=10 patients in each group). **(D)** Stable curve showing the number of trees and an error rate of random forest model for ranking predictors of disease severity in GSE43777 with out-of-the-bag (OOB) of 27.03% and class error of 33,33% and 21,05% for group 1 (DF) and group 2 (DHF), respectively. **(E)** Receiver operating characteristics (ROC) curve of the random forest model with an area under the curve (AUC) of 95,92% for two groups of severity. **(F)** Variable predictors scores plot for classification of dengue infection according to severity. The variables are shown according to minimal depth and number of trees. The color scale bar ranges from 0 to 6 and represents the minimal and maximum minimal depth. The small dark vertical bars represent the mean of minimal depth for each variable. **(G)** Lollipop graph showing biological process gene ontology (GO) terms enriched by the 10 genes classified as most predictors of dengue severity.

Further analysis revealed 20 common DEGs across all DVT (DVT1/DVT2/DVT3) and the NDI data sets – *DDX60, OAS3, ISG15, IFI27, SPATS2L, CXCL10, GBP1, IFIH1, IFI44L, IFIT3, IFI44, HERC5, HELZ2, LAMP3, IFIT1, IFIT2, IFIT5*, and *IFIT2* – (**Figure 8A**). To associate this overlap with disease severity, we used the NDI data set GSE43777. Sixteen (*DDX60, OAS3, ISG15, IFI27, SPATS2L, CXCL10, GBP1, IFIH1, IFI44L, IFIT3, IFI44, HERC5, LAMP3, IFIT1, IFIT5*, and *IFIT2*) of these 20 common DEGs were present and downregulated when comparing late acute with early acute phases from the same disease severity group (DF versus DF or DHF versus DHF) while upregulated when comparing DHF versus DF for late, early, and convalescent phases (**Figure 8B**; **Supplementary Table S21**). Accordingly, PCA based on these 16 DEGs distinguished dengue patients by disease severity (DHF versus DF) and stratified patients mainly at late acute DHF and late acute DF from the other groups (early acute and convalescent patients) (**Figure 8C**). Furthermore, we carried out RF modeling, a machine learning method, to rank these 16 genes as predictors of disease severity when comparing DF and DHF patient groups in the data set GSE43777. The model resulted in an OOB error rate of 27.03%, class error rates of 33.33% for group 1 (DF) and 21.05% for group 2 (DHF), respectively (**Figure 8D**), and a high true positive rate as shown by 95.92% area under the curve of the receiver operating characteristics curve (**Figure 8E**). Among the ten strongest predictors of dengue severity, *IFIT5*, *ISG15*, and *HERC5* (**Figure 8F**) were the three most important variables. These ten genes, mostly downregulated in the late acute phase relative to the early acute phase in GSE43777, enrich biological processes related to interferon and viral immune responses (**Figure 8G**). Of note, although these ten most predictors of dengue severity in the data set GSE43777 were heterogeneously expressed across other data sets containing dengue patients with different disease severity statuses (**Supplementary Figure S6**), their expression pattern was constantly increased at late acute time points of the DVT1, DVT2, and DVT3 data sets (**Supplementary Figures S6A–C**).

### 3.6 Comparative analysis across other viral diseases unveils partially shared interferon-associated signatures

**Figure 9:**
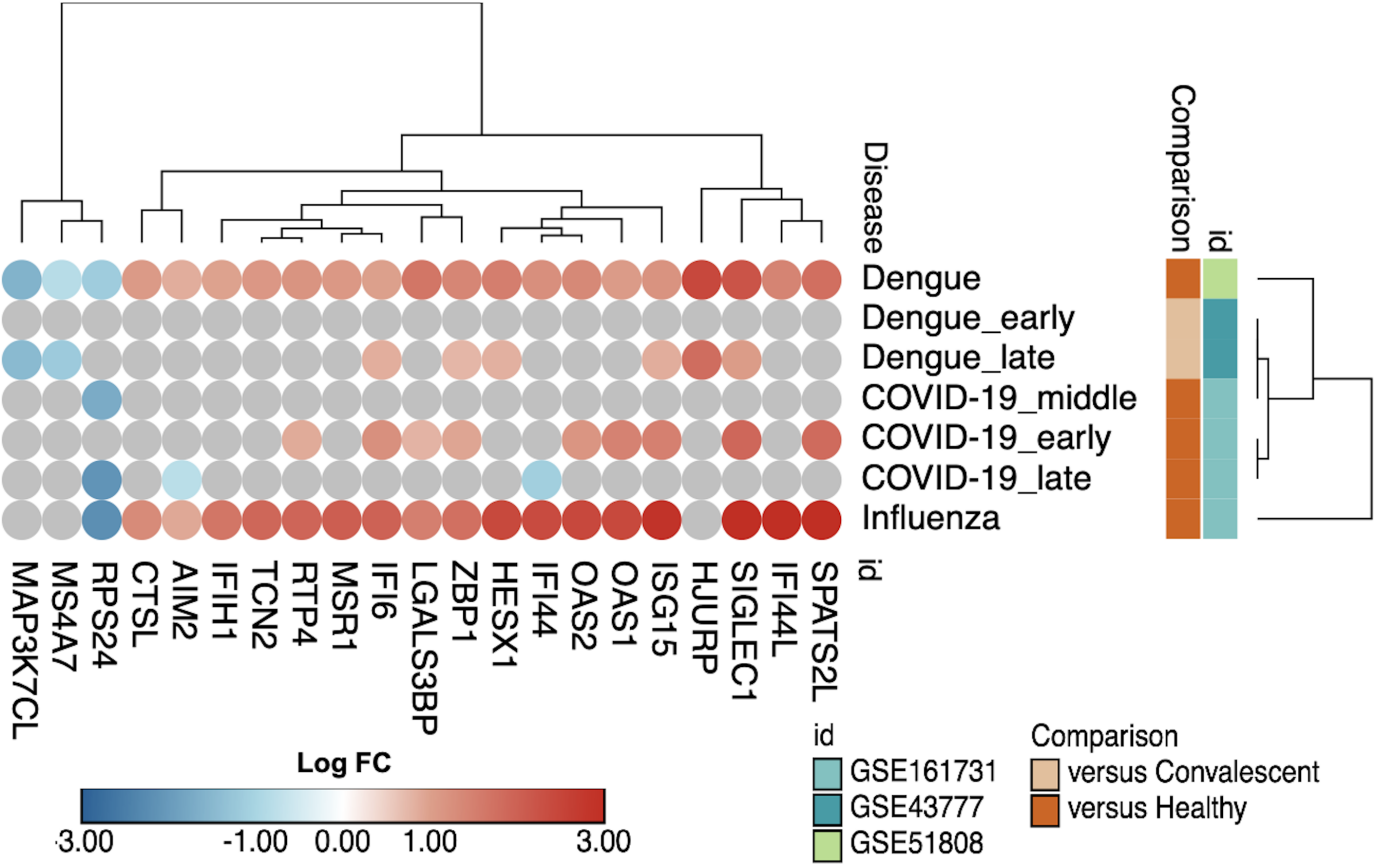
Commonalities in IFN-related genes across different viral infections. Bubble heatmap with hierarchical clustering using Euclidian metric of 16 IFN-related genes comparing dengue and other viral (COVID-19 and influenza) infections.

Identifying an interferon-associated signature marking the overlap of the acute phase in NDIs and the DVTs (Section 3.2.) raised important questions about the specificity of these findings compared to other viral infections. To address this issue, we examined the expression of IFN-related genes across different datasets, comparing dengue, COVID-19, and influenza (**Figure 9**). Several IFN-related genes, such as *SPATS2L, OAS2, ISG15, ZBP1*, and *IFI44L*, exhibited similar patterns of upregulation across both dengue and COVID-19 datasets, while others, such as *AIM2* and *RTP4*, showed distinct regulation.

Moreover, despite the differences in the nature and structure of DENV and influenza virus, patients infected by these two viruses shared the upregulation of several IFN-related genes, such as *ISG15, OAS2, IFI44*, and *IFI44L*. On the other hand, there are notable differences in specific genes, such as *MAP3K7CL* and *MSR1* (**Figure 9**), which were downregulated in dengue but not in influenza. These observations suggest a partially shared IFN signature among dengue, COVID-19, and influenza.

## 4 Discussion

As the demand for safe and efficacious dengue vaccination rises, ongoing development efforts (3), and recent approvals in certain countries (58), contribute to the expanding array of available vaccines. In this context, the results of our analysis, characterizing the overlap of several NDI and DVT data sets, will contribute to future investigations into creating a safe and effective attenuated vaccine (59). Using a systems vaccinology approach, we identified genes that are not only regulated by natural dengue infection but are consistently expressed across infections with attenuated DENV. Among them are DEGs that are upregulated at acute time points across DVT1, DVT2, and DVT3 data sets (e.g., IFIT5, ISG15, and HERC5) and downregulated across NDI datasets in the late acute phase relative to the early acute phase, thus are potential predictors of disease severity being essential players of the anti-viral immune responses, including type I and II IFN signaling (60). Of note, mechanistic studies performed by others support our integrative systems vaccinology findings. For instance, these mechanistic studies demonstrate the critical role of IFITs, ISG15, and HERC5 as mediators of IFN-induced inhibition of viral (e.g., DENV) replication and protection of host cells from apoptosis (61–63). Thus, our work reinforces the potential of attenuated DENV vaccines to solve the growing public health problem caused by DENV infections while suggesting disease severity markers to monitor vaccine efficacy (3) and to assist clinicians to early stratify patients for in depth follow-up.

In addition, secondary dengue infection may promote DHF and DSS due to antibody-dependent enhancement (ADE) by contributing to increased virus entry into host cells (e.g., macrophages), upregulation of IL-10, and inhibition of type 1 IFN production (8, 64, 65). For instance, while the tetravalent attenuated dengue vaccine (Dengvaxia) has shown a high rate of protection for seropositive individuals, i.e., those who already had experienced a previous dengue infection, known as secondary dengue, there was an increased incidence of hospitalization of those who were seronegative when immunized due to Dengvaxia-induced ADE (5). This issue needs to be addressed by future studies and compare immunological signatures of vaccinated individuals who either develop or do not develop severe dengue due to ADE. Such an analysis will contribute to our understanding of this phenomenon and identify biomarkers of ADE for therapeutic exploration.

The NDI/DVT overlap of gene expression involves, among others, the activation of several IFN-associated pathways, which are critical to the anti-viral immune response (60). In addition, we ranked common IFN-associated DEGs by random forest analysis as predictors of dengue severity. IFNs are rapidly induced during viral infections and function as central mediators of the response to DENV (66, 67). The IFN system is essential during the acute phase of DHF in lowering virus production and protecting the host (68). For instance, the binding of IFN-α/IFN-β to their receptors, IFNRA1/IFNRA2, triggers the activation of multiple downstream signaling pathways. For example, the activation of the canonical signal transducer and activator of transcription 1 (STAT1)–STAT2–IFN-regulatory factors (IRFs) signaling complex, which binds to IFN-stimulated response elements (ISREs) in gene promoters, leads to the expression of IFN-stimulated genes (ISGs) (69). In agreement with the NDI/DVT overlap, other transcriptome studies of dengue patients not included here have also identified the IFN system as a significant mechanism elicited in response to DENV (56, 68, 70). In line with our findings, the downregulation of ISGs is associated with reduced production of IFN in DHF and DSS patients compared to DF patients during the acute phase, underscoring the role of IFN-associated pathways for the outcomes of dengue (68, 71, 72). Notably, linear and mechanistic studies (63, 73, 74) corroborate these high throughput investigations.

The shared upregulation of IFN-related genes between dengue, COVID-19, and influenza infections presented here implies a commonality in the host immune response against diverse viral pathogens. This observation suggests a fundamental strategy employed by the host to combat viral infections, emphasizing the well-known importance of the interferon system in antiviral defense (75–77). The implications of these findings for vaccine development are substantial. This shared response could be leveraged to develop broad-spectrum antiviral strategies targeting conserved interferon pathway elements. However, the notable differences in the distinct regulation of genes, highlight the nuanced nature of host-virus interactions, promoting the need for specific approaches. Understanding the unique signatures of each viral infection is crucial for the design of vaccines that elicit precise and compelling immune responses.

While the interferon antiviral pathways may represent an early defense, their complex biological functions set the stage for developing robust adaptive immunity. Genes such as *OAS2, ISG15, AIM2, OAS1, SIGLEC1, IFI6, IFI44L, IFIH1*, and *IFI44*, identified in our integrative systems study, play pivotal roles in orchestrating various aspects of the adaptive immune response. For instance, *OAS2* and *OAS1* has antiviral functions (78), while *ISG15* restricts virus replication (62, 79). *AIM2* activates the inflammasome (80), initiating pro-inflammatory responses crucial for effective antiviral defense. *SIGLEC1* participates in immune cell interactions, facilitating antigen presentation and adaptive immune recognition (81, 82). Interferon-inducible, *IFI6, IFI44L*, and *IFI44*, are implicated in apoptotic modulation and antiviral defense (83, 84). *IFIH1* is a crucial sensor for viral RNA, shaping adaptive responses to RNA viruses (85). *IFIT5* and *HERC5* are known for their roles in IFN-mediated inhibition of viral replication (86). Therefore, the set of relevant genes identified in our study underscore the intricate link between initial antiviral pathways and subsequent adaptive immune processes, pointing out their significance as potential molecular markers for monitoring vaccination efficacy and directing the development of targeted anti-dengue therapies, including immunomodulatory drugs.

In conclusion, our work demonstrates gene expression correlations between natural dengue infection and vaccination and supports ongoing international efforts to develop protective and safe attenuated vaccines against dengue (9, 26). This is highlighted by including the gene expression dataset of Takeda’s live attenuated tetravalent dengue vaccine candidate TAK-003 (Qdenga) that showed efficacy over time against dengue virus of 70% for asymptomatic and mild dengue and lowered the risk of severe cases, including hospitalization and death, by 80–90% (87–89). Nevertheless, additional studies are needed to demonstrate detailed gene expression correlations with viral load and dengue associated NS1 expression during the infection. Since no NS1 or viral load information was available for all datasets, we could not address this issue.

### 4.1 Study limitations

However, this study has limitations, such as the absence of primary and secondary infection information and serotype variations in some datasets included here. Hence, future investigations are required to address the impact of primary versus secondary infections and serotype variations in the overlapping signature between dengue vaccination and natural infection. Furthermore, this study focused on PBMCs, and it would be highly relevant to evaluate the gene signatures in other leukocytes. Notably, DENV activates neutrophils, thereby inducing neutrophil extracellular trap (NET) formation (90, 91). Future studies addressing how the expression of DEGs involved in this process is associated with dengue severity will shedding light on dengue immunopathology. Additionally, while our study suggests a set of DEGs useful to screen potentially efficacious dengue vaccines in early phases of clinical trials, future studies are required to validate at the protein level which DEGs are relevant to monitor anti-dengue vaccines and their capacity to provide long term effective protection, as well as for monitoring different DENV serotypes and individuals with or without pre-existing anti-DENV immunity.

## Data availability statement

All data sets used in this study are publicly available in the GEO database. R codes are available on Github: https://github.com/DesireePlaca/PlacaDR_IntegrativeSystemsVaccinologyDengue.

## Ethics statement

Since available data were utilized for this study, ethical approval was not required in accordance with the national legislation and institutional requirements.

## Author contributions

DP: Conceptualization, Data curation, Formal analysis, Funding acquisition, Investigation, Methodology, Project administration, Resources, Software, Supervision, Visualization, Writing – original draft, Writing – review & editing. DF: Writing – review & editing, Formal analysis, Software, Visualization. AM: Formal analysis, Software, Writing – review & editing. SZ: Formal analysis, Software, Writing – review & editing. JU: Writing – review & editing. GB: Writing – review & editing, Conceptualization. CP: Writing – review & editing. RS: Writing – review & editing, Visualization. IF: Writing – review & editing. PF: Writing – review & editing, Methodology. VR: Writing – review & editing. GC-M: Writing – review & editing. NC: Writing – review & editing. RC: Writing – review & editing. GM: Writing – review & editing. IJ: Writing – review & editing, Visualization. VC: Writing – review & editing. LG: Writing – review & editing. LR: Writing – review & editing. HO: Writing – review & editing. LS: Writing – review & editing, Visualization. OC-M: Writing – review & editing, Conceptualization, Funding acquisition, Investigation, Methodology, Resources.
