# Supplementary material for: Immunological signatures unveiled by integrative systems vaccinology characterization of dengue vaccination trials and natural infection
Source: Front Immunol. 2024 Feb 20;15:1282754. doi: 10.3389/fimmu.2024.1282754 (PMC10912564; doi:10.3389/fimmu.2024.1282754)
Supplement: Supplementary file 1 [file Image_1.pdf]

Supplementary Figure 1

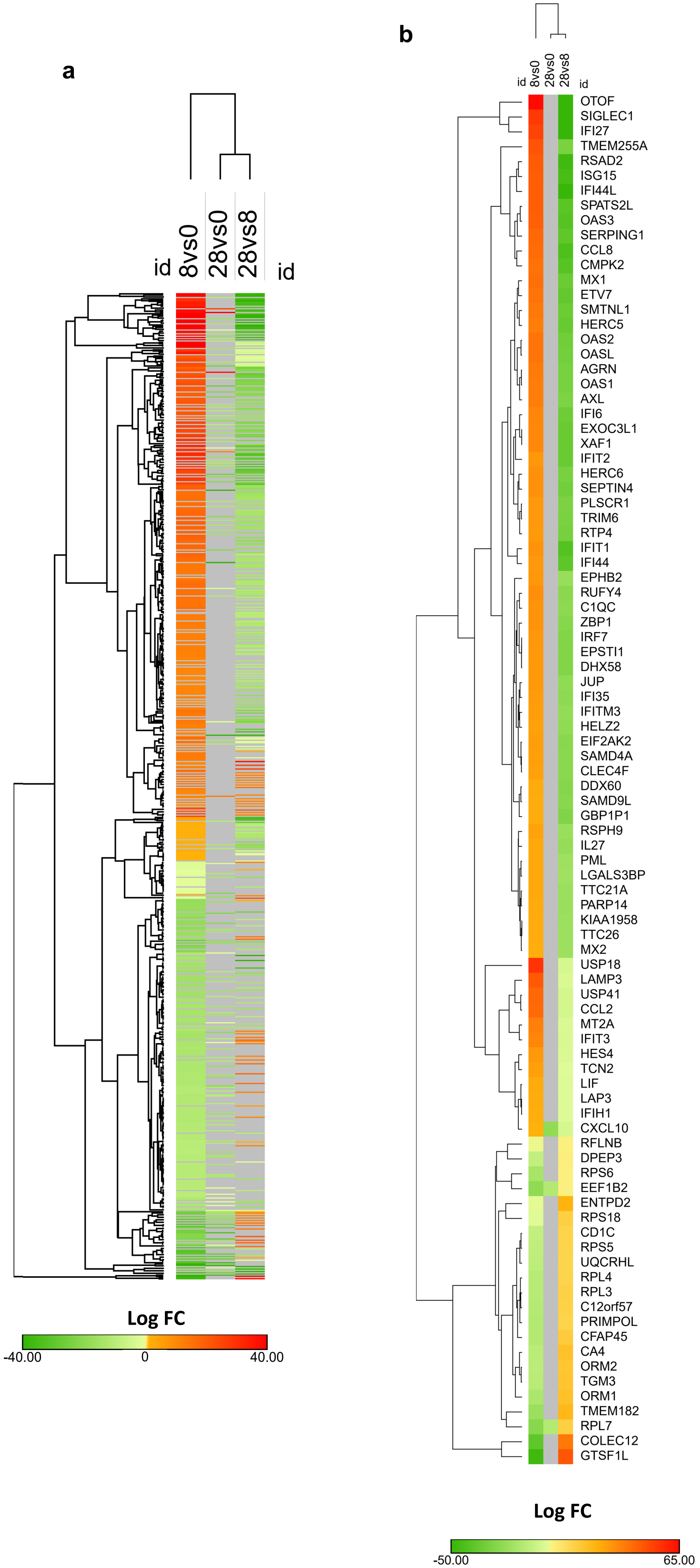

Supplementary Figure 1. Transcriptional clustergrams of different expressed genes during dengue attenuated infection (dataset 152255; 8d *vs.* 0d; 28d *vs.* 0d; 28d *vs.* 8d). (a) Heatmap showing differently expressed genes pattern (793 unique genes) between the time-points sampled (b) Heatmap for the top 70 top up and the down regulated genes filtered on 8d vs. 0 comparison. Clustergrams were obtained from Morpheus and expressed level expressed in log FC, differentiated by colors.

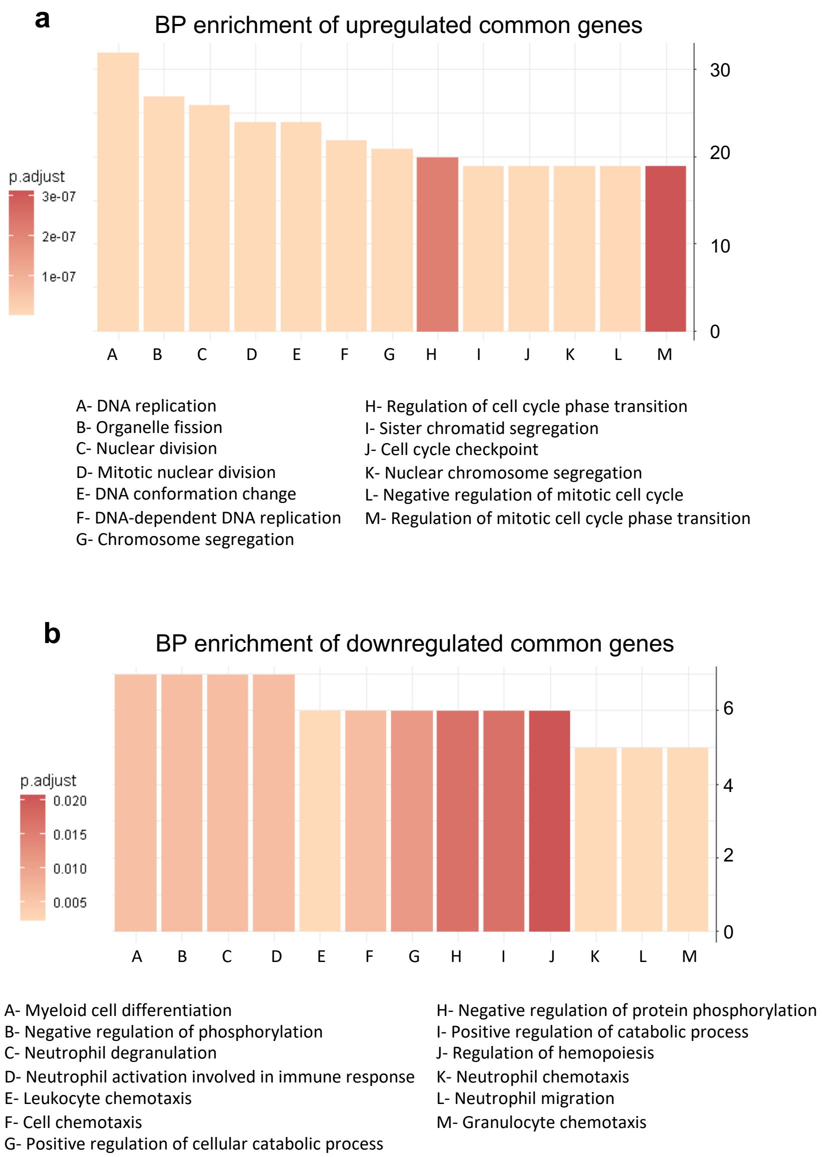

Supplementary Figure 2. Biological processes enriched by the common differentially expressed genes between the dengue acute phase in natural infection. Barplot of biological processes (BPs) enriched by the (**a**) 164 common upregulated and (**b**) 48 common downregulated DEGs during the acute phase (**Suppl.Tables S7-8**). The bar color denotes the adjusted p-value of enriched pathways. Graphs were created using the Cluster Profiler R package.

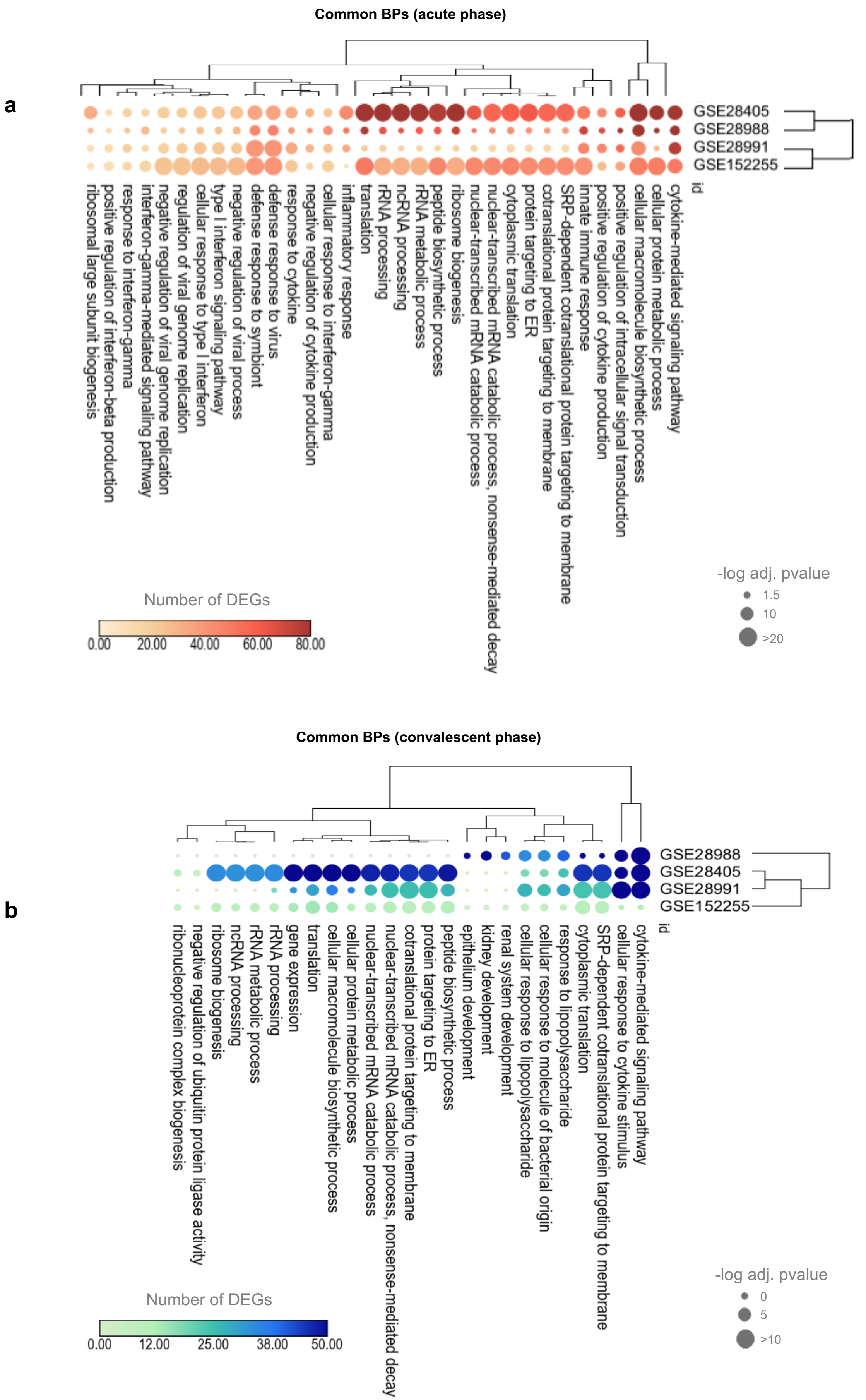

Supplementary Figure 3. Common enriched biological processes between NDI and DVT1 during acute and convalescent phases. (**a**-**b**) Bubble heatmaps with hierarchical clusters by Euclidian distance of common biological processes (BP) in at least two data sets across NDI (GSE28405, GSE28988, GSE28991) and DVT1 (GSE152255) data sets during (**a**) acute (days 4-8 versus day 0-3) and (**b**) convalescent (days 15-28 versus days 0-3) phases after infections. Bubble sizes are proportional to the -log10 adjusted p-value and colors according to the number of DEGs by BP term across the data sets. The complete lists of significant enriched BPs are provided in **Suppl. Tables S14 and S15**.

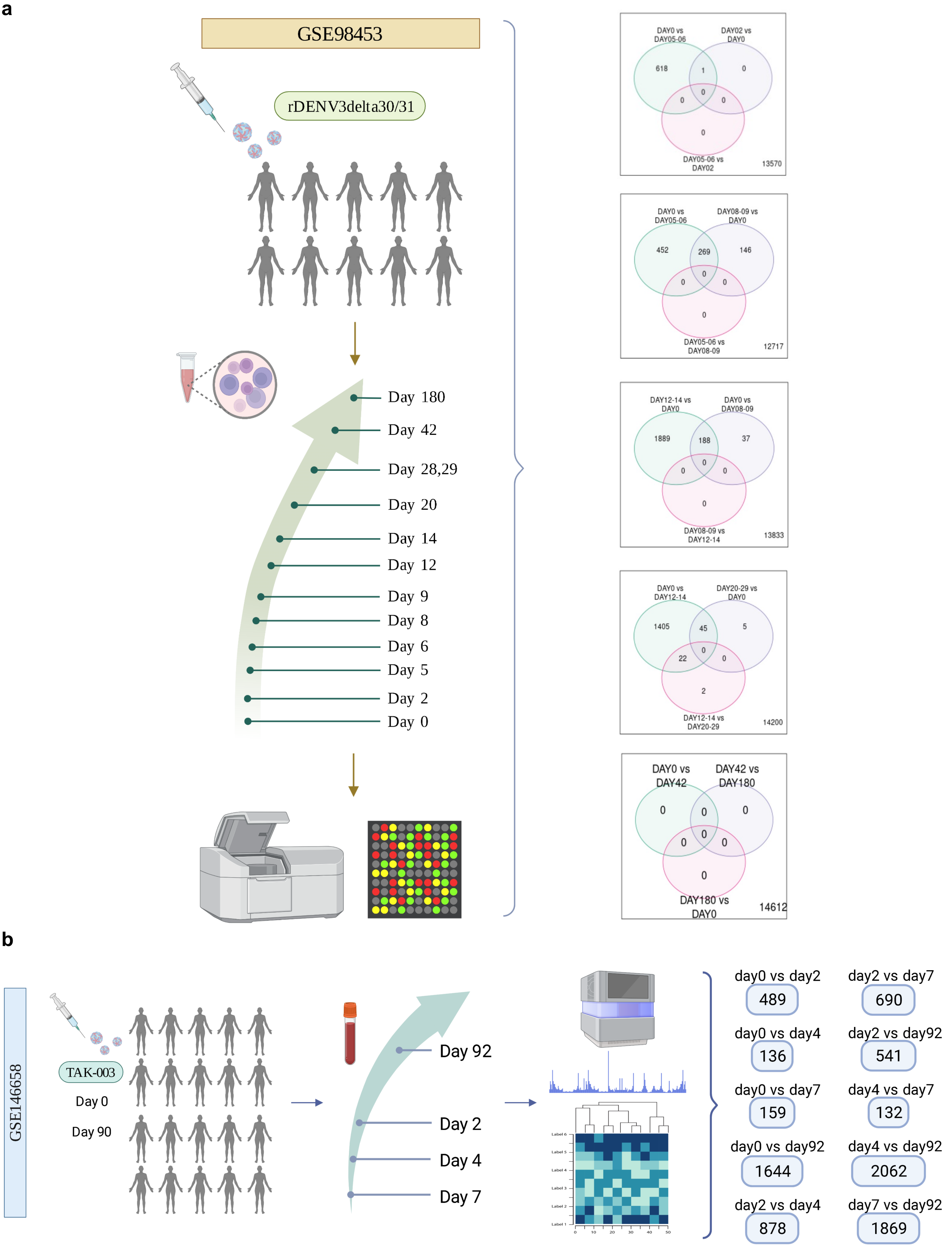

Supplementary Figure 4. Validation data sets resume. (**a**) GSE98053 and (**b**) GSE146658 vaccine trial design schemes, showing the number of patients, days of sample collection, and the number of differentially expressed genes (DEGs) for each comparison.

a

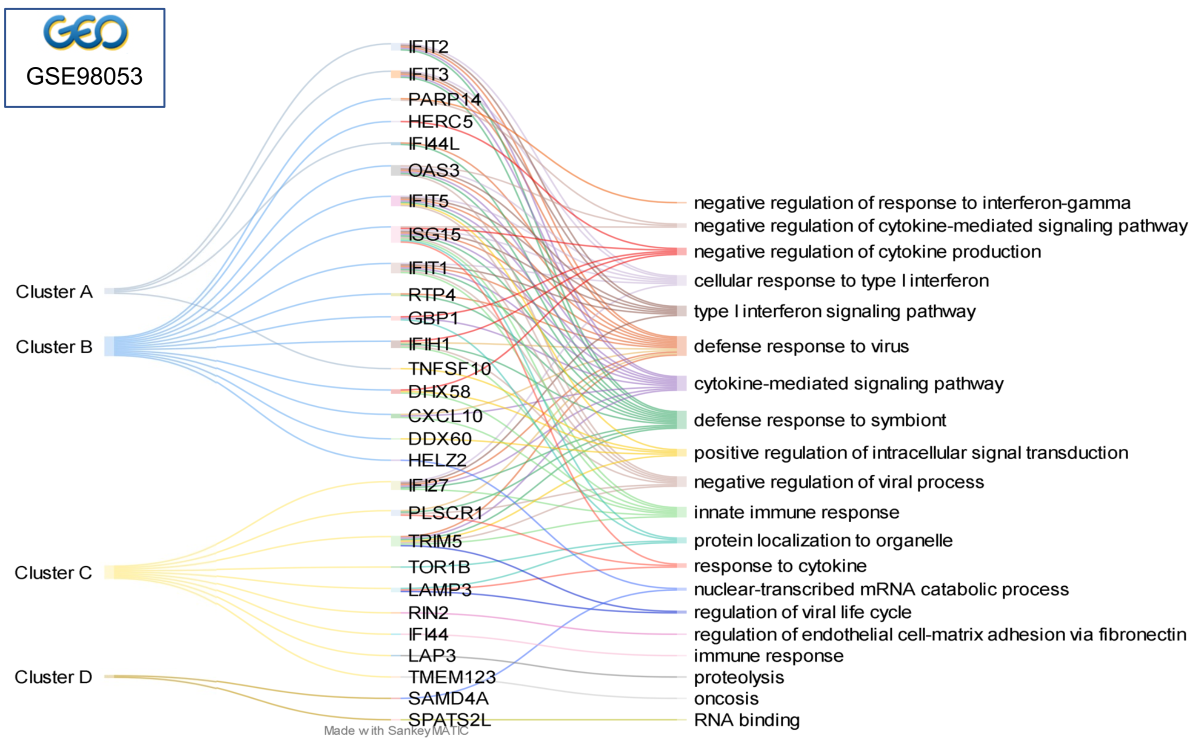

b

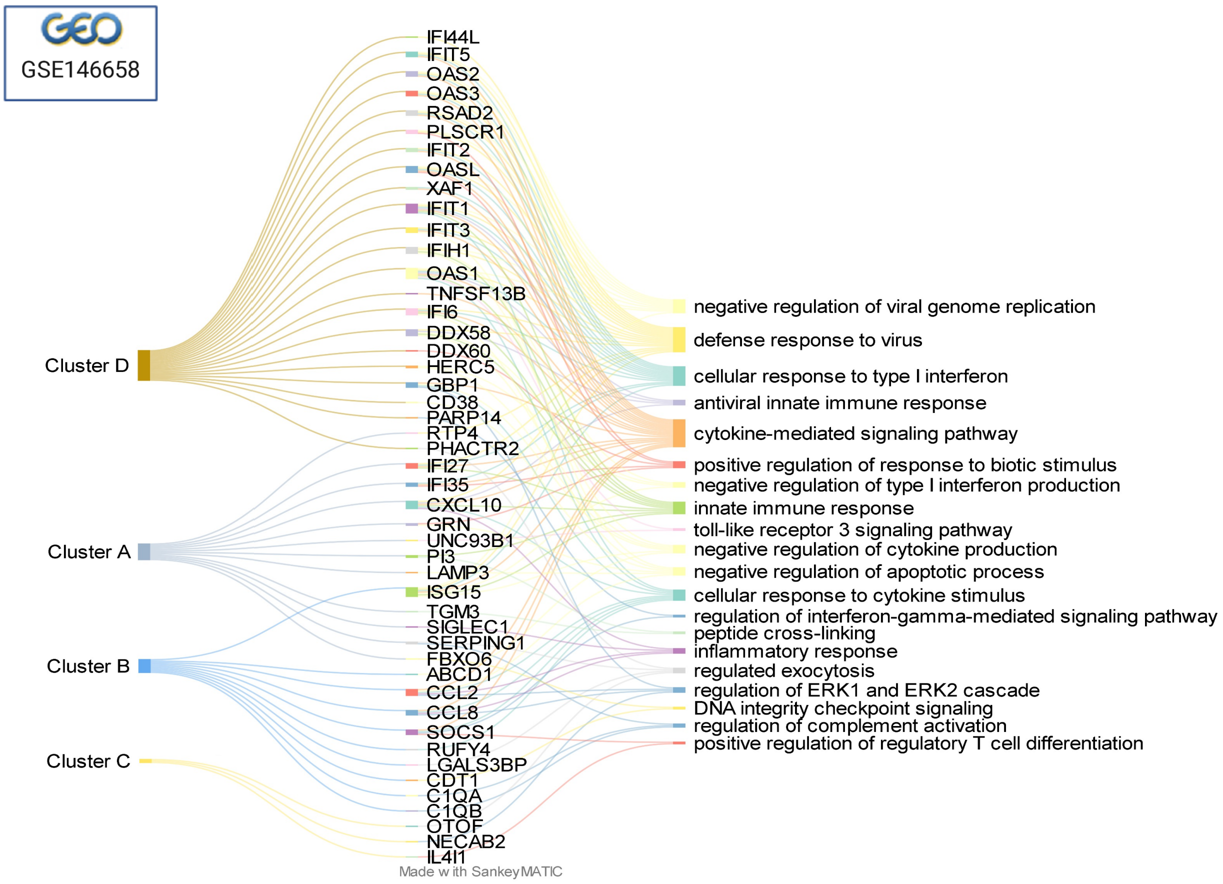

Supplementary Figure 5. Enrichment of genes overlapping in NDI/DVT1 and additional vaccine trials DVT2 and DVT3. (a-b) Alluvial diagrams of 28 common genes from NDI/DVT1-DVT2 (**a**) and 60 genes from NDI/DVT2-DVT3 (**b**) separated by clusters, showing their functional enrichment. BPs were obtained using the enrichR[4] web tool. Complete enrichment tables are available in **Suppl. Tables S17 and S19**.

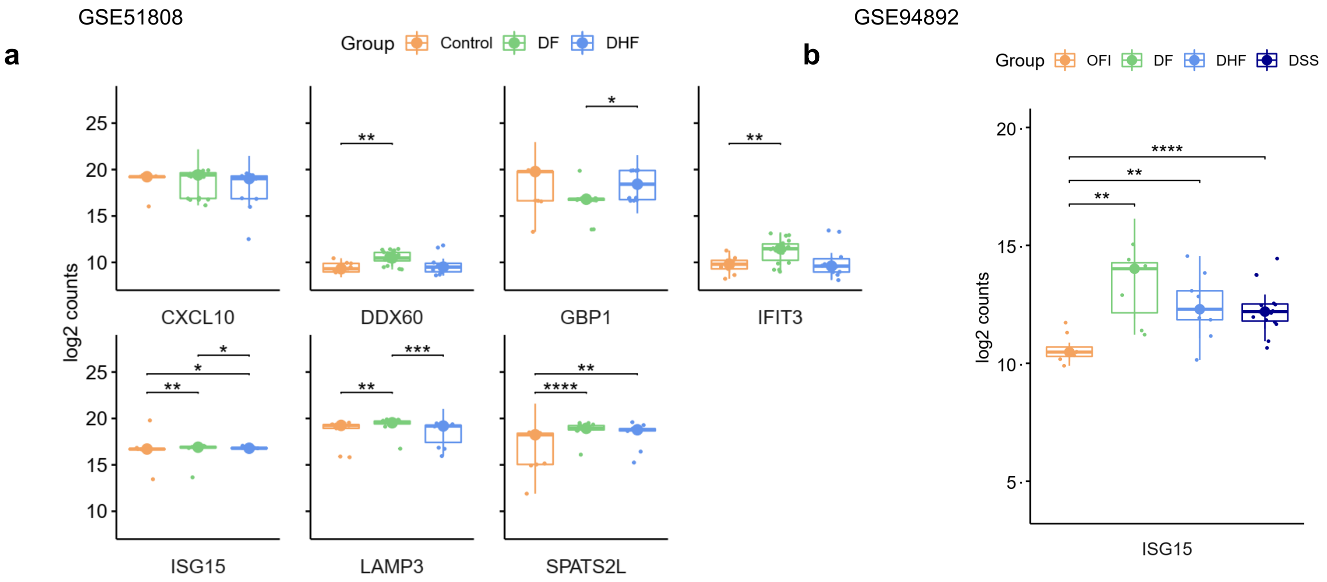

Supplementary Figure 6. Expression pattern of genes classified as most predictors of dengue severity across disease severity groups from other data sets. (**a-b**) Boxplots showing log2 counts of variables classified as predictors for disease severity in the random forest model from groups of the natural dengue infections (NDI) data set (**a**) GSE51808 (Control, DF, and DHF, n=9, n=18, and n=10, respectively) and (**b**) GSE94892 (OFI, DF, DHF, and DSS, n=8, n=7, n=9, and n=15, respectively). All statistics were obtained by wilcoxon.test: * p value < 0.05; ** p value < 0.01; *** p value < 0.001; **** p value< 0.0001. DF: Dengue Fever; DHF: Dengue Hemorrhagic Fever; DSS: Dengue Shock Syndrome; OFI: Other Febrile Illness; Control: healthy controls

Supplementary Figure 7

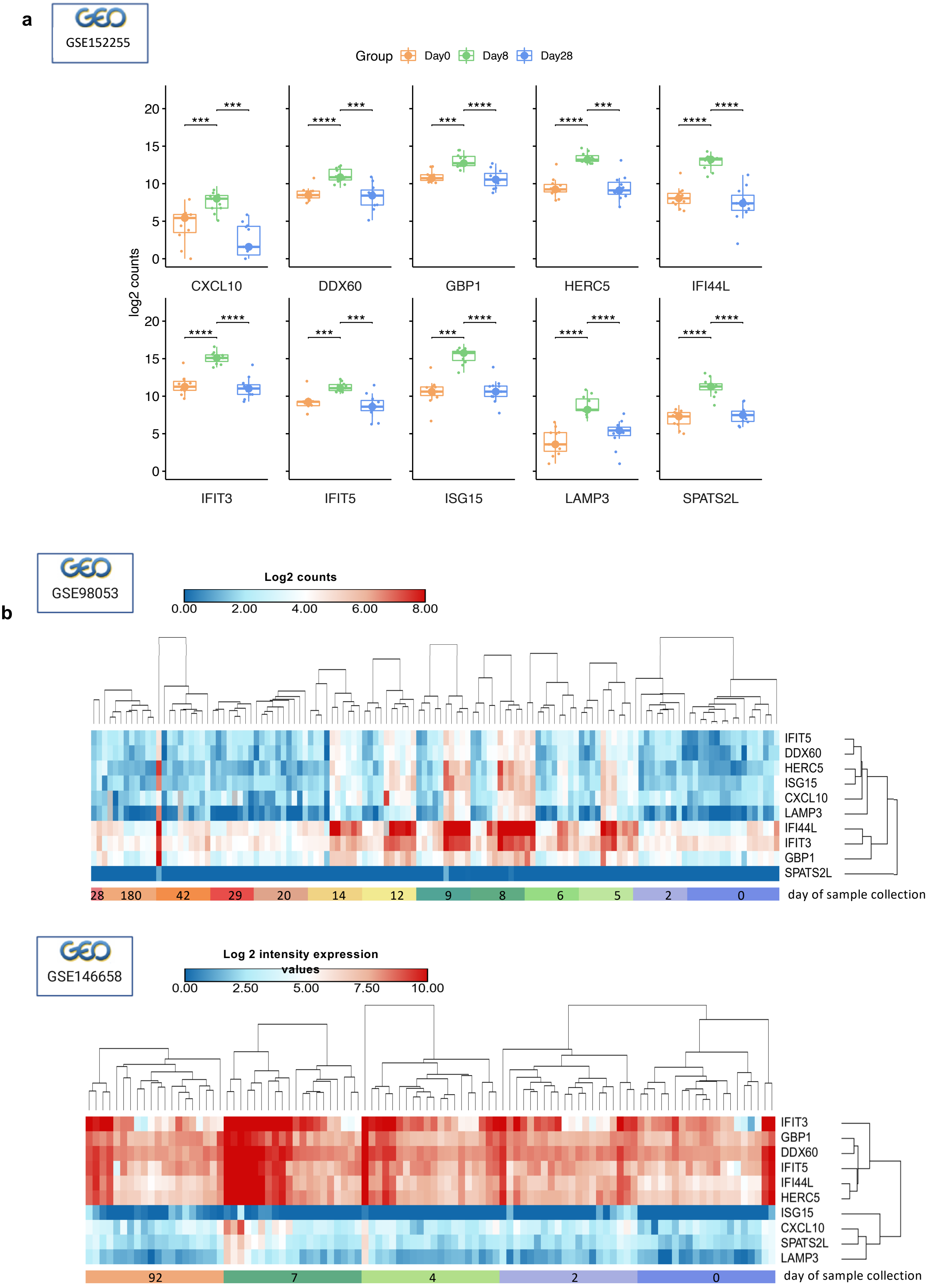

Supplementary Figure 7. Increased expression pattern of 10 genes classified as most predictors of dengue severity at acute time points across all DVT data sets. (**a**) Boxplots showing log2 counts of the 10 genes classified as most predictors for disease severity at three time points in the vaccine-induced response from the DVT1 (GSE152255) data set. Statistical differences were obtained by Wilcoxon.test, using "lemon" and "ggpubr" R packages: * p value < 0.05; ** p value < 0.01; *** p value < 0.001; **** p value< 0.0001. (**b-c**) Heatmaps of expression values of the same 10 genes as in (a) at different time points from the vaccine trials data set (**b**) DVT2_GSE98053 and **(c)** DVT3_GSE146658. Hierarchical clustering was performed using the Euclidean distance metric.
